# Prevention of anemia in children through the consumption of a blueberry and quinoa drink enriched with iron as part of a nutritional program

**DOI:** 10.3389/fnut.2025.1639894

**Published:** 2025-09-26

**Authors:** Cinthya Neglia-Cermeño, Susana Edita Paredes-Díaz, Nancy Soto-Deza, Jaime Bazán-Cabellos, Nélida Milly Otiniano, Jorge Luis Díaz-Ortega, Luz Angélica Castro-Caracholi, Juan Ernesto Valdiviezo-Campos, Victoria Ramos Torres, Carol Abanto-Quiroa, Mariana Zariquiey-Rubio, Karla Nazario-Terrones, Gissela Esteban-Dionicio

**Affiliations:** ^1^Universidad César Vallejo, Escuela de Nutrición, Trujillo, Peru; ^2^Universidad César Vallejo, Escuela de Medicina, Piura, Peru; ^3^Universidad César Vallejo, Institutos y Centros de Investigación, Trujillo, Peru; ^4^Danper, Gerencia de Salud, Trujillo, Peru; ^5^Universidad César Vallejo, Escuela de Medicina, Trujillo, Peru; ^6^Danper, Gerencia Técnica, Trujillo, Peru

**Keywords:** anemia, nutritional program, hemoglobin, ferritin, anthropometric indicators

## Abstract

**Objective:**

To demonstrate the preventive effect against anemia in children by the consumption of a blueberry and quinoa drink enriched with iron as part of a nutritional program.

**Method:**

The nutrition program involved 42 children aged 1 to 6 years. They were the children of workers in an agro-industrial company in the city of Trujillo, where they received a blueberry and quinoa drink enriched with iron in the form of ferric pyrophosphate (BQDEI), the content of which in a 200 mL bottle provides 14 mg of Fe. For children without anemia the dose was 1 bottle per day and for children with mild anemia the dose was 2 bottles per day for a period of 6 months. Educational and demonstration sessions were held for parents or caregivers regarding proper food handling, safe water consumption, the importance of personal hygiene and the benefits of a balanced diet rich in iron. In addition, nutritional status evaluation, parasitosis control and ferritin and hemoglobin measurements were performed at the beginning, third and sixth month during the development of the nutritional program.

**Results:**

50% of the children with short stature reached normal height, and 80% of the children’s weight/height went from overweight to normal, and these changes were significant. Ferritin concentration in the initial, intermediate and final phases was 40.67 ± 25.20 ng/ml, 49.37 ± 22.41 ng/ml and 54.19 ± 35.38 ng/ml, respectively, showing a significant increase. The hemoglobin concentration was 11.71 ± 0.91 g/dl, 11.83 ± 0.88 g/dl and 11.99 ± 1.03 g/dl, respectively, and the increase was not significant.

**Conclusion:**

It was demonstrated that the consumption of BQDEI within a nutritional program contributed to the prevention of anemia in children, by improving hemoglobin and ferritin concentrations. Therefore, it could be implemented in other areas where children are more vulnerable to anemia.

## 1 Introduction

Anemia is a worldwide health problem characterized by a low hemoglobin (Hb) blood concentration. This nutritional disorder can occur at any stage of life. However, women and preschool children are the most vulnerable (1). It occurs in populations of various regions, genders and ages. Anemia is most prevalent in developing countries (2), and is a health problem of particular concern in third world nations. In this sense, the World Health Organization (WHO) specifies Hb levels below 11 g/dl for this pathology (3) and that, worldwide, it occurs in approximately 40% of children aged 6 to 59 months (4). In 2023, in Peru, anemia affected 43.1% of children aged 6 to 35 months. The highest incidence was in rural areas (50.3%) compared to urban areas (40.2%). This problem was more prevalent in the departments of Puno, Ucayali, and Madre de Dios, at 70.4, 59.4, and 58.3%, respectively (5). In the case of La Libertad, anemia varied from 17.0% in 2001 to 21.1% in 2024 (6). The age group with the highest level of anemia corresponds to children between 6 and 18 months of age between 54 and 65% (7).

Childhood anemia is a multifactorial condition with a high global prevalence, particularly among children under 3 years of age (47%), with higher rates observed in low-income countries (70%) and among disadvantaged indigenous populations (30%). It is associated with deficiencies in the nutrients iron, folate and vitamin B12, as well as intestinal parasitosis, chronic infectious diseases, premature birth, low birth weight and poor breastfeeding practices involving the consumption of cow’s milk (8–15). Conversely, exclusive breastfeeding and a high socioeconomic status act as protective factors (16). Genetic causes affecting Hb synthesis and blood cell formation are also recognized (17). This condition is also related to variables such as age, sex, race, illiteracy (18–21), other nutritional deficiencies such as folate and vitamin B12 (14, 22), and poor sanitary conditions, including limited access to clean drinking water (23, 24), leads to infection by parasites and bacteria that compete for iron in the intestine (22, 25). Finally, the availability of medical services, the family environment, and childcare practices also influence its prevalence (1, 26).

International agencies such as WHO recommend a combination of four basic strategies including iron supplementation, nutritional education, fortification of foods with iron compounds, and control of parasitic and infectious diseases to prevent and control anemia (27). Likewise, the nutrition education applied should be easily adapted to the socioeconomic status, dietary habits and traditional food resources available locally (28, 29).

Nutritional programs play a crucial role not only in the treatment of anemia, but also in its prevention and long-term management, as they provide a comprehensive strategy to address nutrient deficiencies and improve the overall health of children. This is why Soncco-Sucapuca et al. (30) applied a nutritional education program in parents of Peruvian children, achieving a significant decrease in anemia in their children through the consumption of iron-fortified bread, the promotion of healthy practices and the improvement of the level of knowledge about iron deficiency anemia. Likewise, Reyes et al. (31), evaluated the impact of a community intervention program, which included mass campaigns and group workshops for children and parents on issues related to anemia and malnutrition, and showed that the program had a positive influence on the reduction of anemia and malnutrition in children.

It is very important to treat anemia in children under 3 years of age, as it can cause children to become severely and long-term ill, resulting in mental decline, learning and concentration difficulties, poor school performance, stunted growth, impaired motor skills, slow language development, behavioral problems, low immunity, fatigue, lethargy, vulnerability to infections, and an elevated risk of mortality and morbidity (14, 18, 32).

Iron compounds used in food fortification are classified as inorganic salts, organic salts, and chelates. Ferrous sulfate, a low-cost, highly soluble inorganic salt, is widely used, although its high reactivity can affect the sensory properties of certain products. Organic salts (gluconate, lactate, citrate) are also soluble and reactive, while other less soluble salts (saccharate, succinate, fumarate) have good gastric bioavailability but are not suitable for flours due to their tendency to rancidity (33). Ferric pyrophosphate (FPP), an iron compound insoluble in water, is commonly incorporated into cereal products due to its favorable sensory stability. However, its limited gastrointestinal absorption poses a challenge for effective iron delivery (34).

Micronization techniques have been shown to enhance its bioavailability without compromising the organoleptic or functional properties of the food matrix. In contrast, iron–amino acid chelates exhibit superior absorption and minimal reactivity, positioning them as a promising alternative for nutritional supplementation (35).

Hb concentration and serum ferritin are key biomarkers of iron metabolism, as Hb, the main component of erythrocytes, depends on iron for the synthesis of the heme group, a process regulated by erythropoietin in the bone marrow (36).

Blueberries are fruits that, in addition to providing antioxidants, also provide iron, which varies between 2.8 and 8.9% of the daily requirement of this mineral in children, according to different reports (37–39). The protein content of blueberries ranges from 0.32 to 0.62% per 100 g of product (40).

In the present study, a blueberry-based drink has been developed, enriched with iron using FPP, which is also stabilized with amino acids. The addition of quinoa, an Andean grain that provides between 19.34 and 20.90 g per 100 g of product (41), increases the protein content.

Based on the above, the hypothesis was proposed that the administration of a nutritional drink made from blueberries and quinoa enriched with iron, as part of a dietary intervention program, generates a significant increase in Hb and serum ferritin levels in children.

This is why, this research aims to evaluate the prevention of anemia in children through the consumption of a blueberry and quinoa drink enriched with iron within a nutritional program as an option to counteract the rate of childhood anemia.

## 2 Materials and methods

### 2.1 Selection of the participants

The participants were children of workers of an agro-industrial company in the city of Trujillo. The selection criteria included children with mild anemia (14%) and children without anemia (86%), and with an age range of 1–6 years. Also, children without parasitosis and those who had an antiparasitic treatment prior to the beginning of the study were considered. On the other hand, those children with incomplete laboratory data, non-tolerance to the product, parental decision not to continue in the study were excluded.

For the sample size calculation, the following formula was considered (42):


$$n=\frac{{\left(\mathrm{Z\alpha}/2+\mathrm{Z\beta}\right)}^{2}}{d^{2}}$$

In this formula, the value of Zα/2 was set at 1.96; a statistical power of 0.9 was considered, resulting in a Zβ value of 1.28. Cohen’s d was 0.55, derived from the difference in mean Hb concentrations and a standard deviation equal to the square root of the sum of the sample variances divided by two (43). The data corresponded to the period before and after the implementation of a nutritional program developed in Puno (30). The calculated sample size “n” was 35, but a 20% increase was applied to account for potential attrition, resulting in a final sample of 42 participating children.

**Figure 1:**
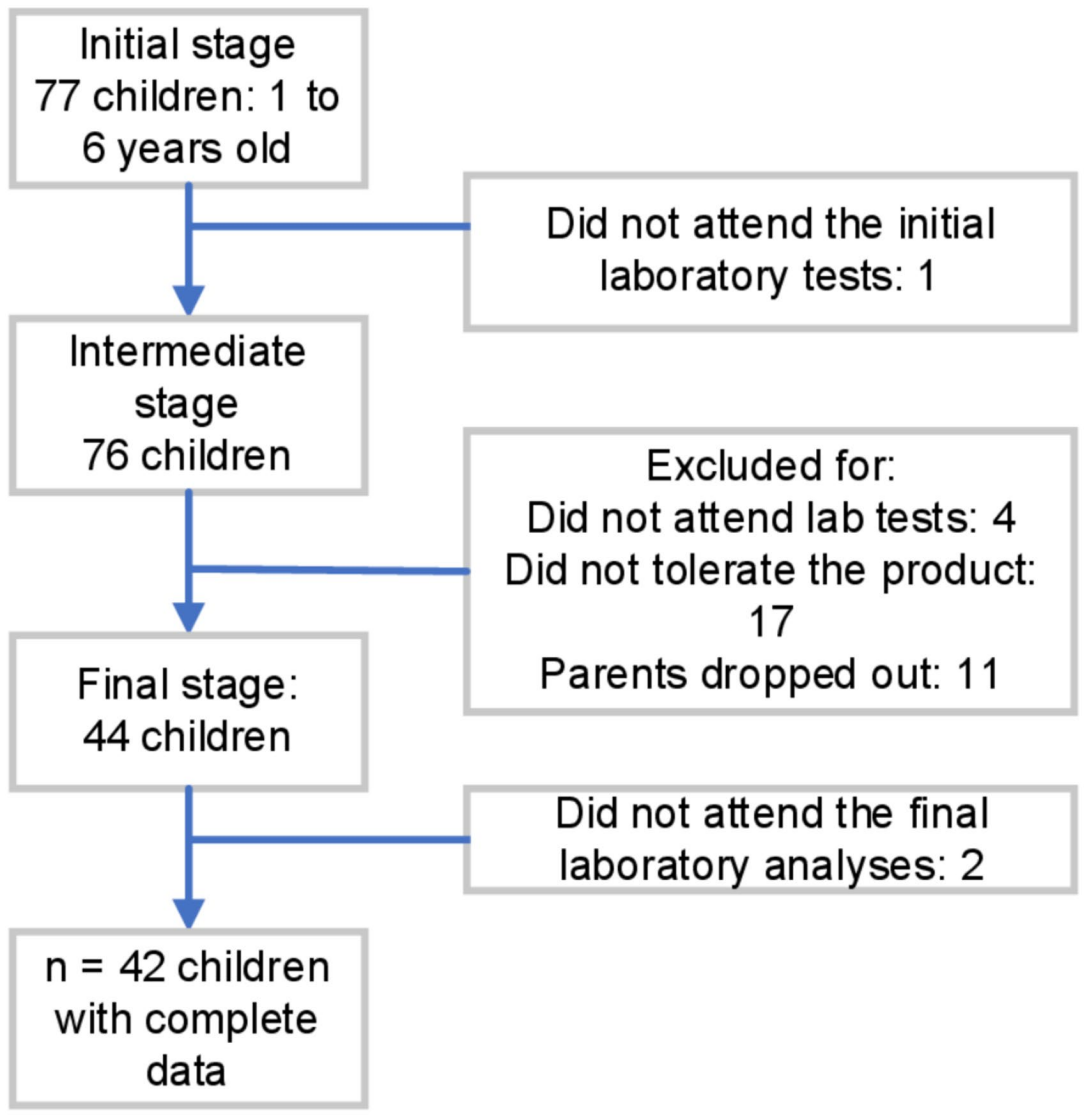
Flow chart of inclusion and exclusion criteria of individuals.

Initially, 77 children were considered eligible and monitored across three stages: initial, intermediate, and final. Among them, those who did not attend the laboratory tests for Hb and ferritin were excluded, one in the initial stage, four in the intermediate stage, and two in the final stage of the study. Additionally, 17 children were excluded during the intermediate stage for not consuming the product due to lack of acceptance, and 11 children were excluded because their parents withdrew from the study for external and personal reasons. Therefore, the final sample for analysis consisted of 42 children (Figure 1).

### 2.2 Design of the study

The research was designed as a pre-experimental study for a period of 6 months, in which the children entered a nutritional program that included the provision of the BQDEI. The study period was chosen because depleted body iron stores require no less than 4 months of iron supplementation for proper recovery (44).

### 2.3 Characteristics of the drink and administration

The BQDEI was manufactured at the processing plant of the agro-industrial company located in the province of Trujillo, La Libertad region. Glass bottles with a capacity of 200 ml (205 g) were used for packaging, in compliance with current regulations and standards (45). In addition, it had a sanitary registration (RSA P2761222N LADNTU) issued by the General Direction of Environmental Health and Food Safety (DIGESA, from Spanish initials) and official technical validation of Hazard Analysis and Critical Control Points in accordance with Directorial Resolution, file N°24,099-2024-CH (46).

A sensory evaluation of the product was carried out with the aim of determining its acceptability among children. The methodology was based on ASTM E2299-13 (47), Standard Guide for Sensory Evaluation of Products by Children and Minors, which allows the use of facial scales in children from 3 years of age. This guide presents examples of scales from 3 to 9 points, without establishing a mandatory format, provided that it is understandable to minors. In this study, a 5-point facial scale was used, selected for its simplicity and clarity, which facilitates interpretation by children and improves the reliability of responses (Supplementary Instrument S1). Twenty-eight children between the ages of 9 and 10 participated, all with written permission from their parents or guardians. This age group was chosen because of their greater cognitive development, verbal ability, and understanding of scales, which allowed for more accurate sensory data to be obtained. Before the evaluation, a familiarization session was held with the terms and expressions of the scale, using visual examples and explanations adapted to the age of the participants. The product received an average rating of 4.29 points, with 89% favorable responses, demonstrating high acceptance. Subsequently, the delivery of the product was organized to the parents of children aged 1 to 6 who expressed interest in participating, using an individualized schedule that ensured orderly and personalized distribution.

The drink was fortified with iron using FPP stabilized with amino acids and sourced from an international supplier specializing in food-grade supplements. This innovative iron source offers high bioavailability and stability under demanding processing conditions, as well as sensory compatibility. These properties make it suitable for use in fortified foods and drinks. The formulation enables higher concentrations to be achieved without altering the organoleptic properties and reduces iron reactivity, enhancing intestinal absorption while minimizing adverse effects such as gastric discomfort or a metallic aftertaste. It also ensures uniform distribution in liquid matrices (48).

As illustrated in the product processing flow diagram in Supplementary Figure S1, FPP was added during the ingredient incorporation stage of smoothie preparation, at the same time as the quinoa, blueberry and other components.

The BQDEI contains the equivalent of 7.0 mg of iron in 100 ml of the drink. In an external laboratory (accreditation certificate D PL146020100 according to DIN EN ISO/IEC 17025:2018), iron analyses were performed during product development and over the course of the product shelf life, with the iron content remaining stable. The nutritional composition of the BQDEI for 1 bottle (200 ml) is detailed in Table 1.

**Table 1:** Nutritional composition of the BQDEI.

Nutritional information			
Serving size:	1 bottle (200 ml)		
Servings per container:	1		
	Quantity per 100 g	Quantity per serving	%VD^*^ serving
Energy (kcal/kJ)	30/129	61/257	3%
Total Fat (g)	0.2	0.4	1%
Saturated fat (g)	0.0	0.0	0%
Trans fat (g)	0.0	0.0	-
Cholesterol (mg)	0.0	0.0	0%
Sodium (mg)	1.2	2.4	0%
Available carbohydrates (g)	6.7	13.4	5%
Total sugars (g)	3.5	7.0	-
Dietary fiber (g)	4.8	9.7	35%
Proteins (g)	0.5	0.9	2%
Iron (mg)	7.0	14.0	64%

* Percent Daily Values are based on a diet of 2,000 kcal (8,370 kJ) according to Codex/FDA.

Daily values may be higher or lower depending on their energy needs.

For children without anemia the dose was 1 bottle per day and for children with mild anemia the dose was 2 bottles per day (morning and afternoon).

### 2.4 Nutritional program

In addition to the consumption of the BQDEI, the nutritional program included a series of activities such as: pediatric care, nutritional care, detection and treatment of parasites. Anemia detection through Hb and ferritin analysis, as well as educational sessions on balanced nutrition and iron-rich foods, and home visits. One of the important aspects of the nutritional program was the follow-up based on a schedule of visits, which made it possible to adjust intervention strategies in real time through direct observation of the children’s eating behavior during the intervention stage. One of the activities carried out was personalized counseling, which used the plate of good eating to specify the appropriate portions and quantities that the child should consume during the day (49). These details ensured that families receive guidance tailored to their needs, thus promoting sustainable eating habits.

In order to optimize its impact, the program was based on two fundamental theories. According to Albert Bandura’s Social Learning Theory (50), parents acquire appropriate behaviors through observation during home visits and practical demonstrations. The positive reinforcement facilitated the consolidation of these habits; The Transtheoretical Model of Behavior Change (51) was used within the study, helping families to move through the stages of change, from awareness of the importance of iron to the sustained adoption of healthy practices.

Educational and demonstration sessions for parents/caregivers included training on proper food handling, safe water consumption, the importance of personal hygiene, and the benefit of an iron-rich diet. Parents/caregivers were educated about high, medium and low iron foods and how they should be combined.

Ongoing nutritional counseling and home visits made it possible to adapt the consumption of the BQDEI within their daily diet through practical demonstrations at home to parents, guiding and reinforcing the consumption of the BQDEI and the recommended intake for each child according to their needs during the nutritional intervention.

### 2.5 Determination of the socioeconomic characteristics of the children’s families

To identify the socioeconomic characteristics of the children’s families, a data collection form was used according to Pacovilca et al. (52), which was answered by the child’s father, mother or caregiver and included data on family income, occupation, marital status, education level, origin, number of members and basic services (Supplementary Instrument S2).

### 2.6 Anthropometric nutritional evaluation

The anthropometric nutritional evaluation was carried out by nutritionists and nutrition facilitators in two stages (initial and final) with the application of the technical guide for the anthropometric nutritional evaluation of children aged 0 to 11 years (53). For children under 5 years of age, the weight/height parameter with categories of severe malnutrition, malnutrition, normal, overweight and obesity and height/age with categories of severe low, low, normal and high height were used for the nutritional diagnosis according to sex. For children over 5 years of age with the diagnosis of BMI/age and height/age according to gender. The evaluation protocols of the anthropometrist’s manual and the MINSA tables (54, 55) were applied to establish normal values and refer to their anthropometric conditions (Supplementary Tables S1–S3).

### 2.7 Evaluation of dietary patterns

We used a card proposed by Arimond et al. (56) in charge of the nutrition facilitators; it considered as indicators the consumption of 6 food groups: cereals, roots and tubers; legumes and nuts; dairy products; meat and red offal and finally fruits and vegetables according to the frequency of daily consumption, 1, 2, 3, 4, 5 and 6 times/week, both at the initial and final moment of the nutritional program, with instruments such as the frequency of consumption of food groups, both at the initial and final moment of the nutritional program (Supplementary Instrument S3). Likewise, the 24-h recall method was applied as described in the nutritional diagnosis protocol of the Association of Nutritionists of Peru, which is governed by MINSA regulations (54). This protocol is based on collecting as much data as possible about the food and drinks consumed the previous day, including type, quantity and method of preparation. Thus, the accuracy of the information collected from the responses to the food pattern questionnaire is reinforced by recalling the short-term memory.

### 2.8 Evaluation for the presence of intestinal parasites

Stool samples were collected using coded plastic containers given to the mothers or caregivers of the children with detailed instructions for serial collection and storage at three different times every 24 h starting at 7 am of the day. Stool samples contaminated with water or urine were rejected. Stool samples were kept in cold chain (57), until they were transferred to the Home Safety laboratory of the Policlínico de Alta Gracia in the city of Trujillo, where they were analyzed.

To detect the presence of intestinal parasites, feces were examined by direct observation with lugol. Positive samples were stored in a plastic tube containing 10% formalin. Single qualitative thick Kato-Katz smears were prepared from each stool sample and examined with a Gretlab microscope, model XSZ159, China at 10X and 40X for the presence of helminths. All participants were given the analyses and the children with positive results underwent a pediatric consultation where they received individualized treatment for parasitosis as appropriate (58, 59).

### 2.9 Hemoglobin and ferritin analysis

The blood samples were collected by venipuncture in vacutainer; 5 ml of blood were drawn aseptically; in purple tubes with anticoagulant K2E EDTA (ethylenediaminetetraacetic acid) and the samples were coded for identification of each participant. The tubes were placed in a cooler box at 15 °C until analysis in the Home Safety Sac laboratory. The blood samples were centrifuged at 3000 rpm for 10 min at room temperature with a Greetmed centrifuge, model GT119-300, China and stored at −20° C.

The Hb concentration was measured using a Horiba ABX SAS hematological analyzer model ABX Pentra XL80, France. For the classification of the diagnosis of anemia according to the specific age of each child at the end of the study, two technical standards issued by MINSA (60, 61) were considered (Supplementary Table S4).

To measure ferritin concentration, the chemiluminescence method (62) was used with the Snibe immunoassay analyzer, model Maglumi 800, China. These analyses were performed at three time points: Basal, at the third and at the sixth month during the development of the nutritional program.

Anemia was defined as a reduction in Hb levels below the reference values specified in Supplementary Table S4. To assess iron status in children, serum ferritin concentrations were measured, applying a threshold of 20 ng/ml. Values below this cutoff were interpreted as indicative of iron deficiency, given their alignment with the plateau phases of other physiological markers. Specifically, reduced mean corpuscular volume suggested hypochromia, while decreased mean corpuscular Hb reflected microcytosis (63).

### 2.10 Statistical analysis

The SPSS statistical program Version 26 was used. For descriptive statistics, frequencies and percentages were used for the different categories of baseline characteristics, anthropometric nutritional status, presence of parasites and dietary patterns. Since the means and standard deviation for ferritin and Hb concentrations did not show a normal distribution, the nonparametric Friedman test was used in the inferential analysis (64), to compare them before, during, and after the implementation of the nutritional program, with a 95% confidence interval and a comparative significance level of 0.05. Cohen’s d was applied to calculate the effect size, considering the ferritin and Hb averages and the standard deviation between the baseline and end of the nutritional program. This effect was corroborated with Kendall’s W to analyze the difference between the initial and final ferritin and Hb medians. Values from 0.2 to 0.49 indicate a small difference; from 0.5 to 0.79, a moderate difference; from 0.8 to 1.29, large (65).

### 2.11 Ethical aspects

The present research has been approved by the Ethics Committee of the Professional School of Nutrition of the Universidad César Vallejo with Report PI-CEI-NUTRITION-2023-001. Ethical principles were considered according to the Declaration of Helsinki (66) in relation to the care of integrity, privacy, confidentiality of personal information and respect for their autonomy. The informed and voluntary consent of the parents was considered, in which the objectives of the study, the procedures to be developed and the information on the risk–benefit of the nutritional program were oriented, in which it was assured that there would be no implication on the physical integrity of the children. The decision of the parent not to continue with the research in special situations or disagreement was also respected, without adversely affecting the researcher-participant relationship.

## 3 Results

### 3.1 Socioeconomic characteristics of the children’s families

According to Table 2, among the most relevant socioeconomic characteristics, it was found that the family income is greater than $ 534.76 in 76.2% of the families, the most predominant occupation of the parents is dependent in 85.7%; the majority are married or cohabiting, and the most prevalent level of education is high school in two thirds of the families. More than three-quarters of these families come from urban areas; all have access to sewage systems, but 11.9% lack potable water and 14.3% do not have electricity service.

**Table 2:** Basal socioeconomic characteristics of children under 6 years of age who consumed the BQDEI as part of a nutritional program.

Characteristics			f	%
Sex	Female		23	54.8
	Male		19	45.2
Family income ($)	Less than 274.06		2	4.8
	274.06–534.76		8	19.0
	More than 534.76		32	76.2
Occupation	Student		1	2.4
	Dependent		36	85.7
	Independent		5	11.9
Marital status	Single		1	2.4
	Married/cohabitant		35	83.3
	Separated/widow		6	14.3
Level of education	Elementary School		6	14.3
	High School		28	66.7
	Higher education		8	19.0
Origin	Rural		10	23.8
	Urban		32	76.2
Number of members	≤4 members		24	57.1
	>4 members		18	42.9
Basic services	Water	No	5	11.9
		Yes	37	88.1
	Electricity	No	6	14.3
		Yes	36	85.7
	Sewage	No	0	0.0
		Yes	42	100.0
Total			42	100.0

### 3.2 Anthropometric nutritional status

Table 3 shows that the proportion of children with normal height/age increased from 85.7 to 95.2%, the most important fact being that two of the four children (50%) with low height managed to reach normal height significantly (*p* < 0.001). Regarding the anthropometric measure weight/height, it was observed that of the five children who started with overweight, 80% managed to reach a normal weight and one child in a state of malnutrition reverted this situation at the end of the study, so the intervention applied was successful (p < 0.001).

**Table 3:** Initial and final anthropometric assessment (height/age and weight/height) in children under 6 years of age during the consumption of BQDEI within a nutritional program.

Height/age initial	Height/age final				Total		Person’s Chi-square	Asymptotic significance (bilateral)
	Low		Normal					
	f	%	f	%	f	%		
Low	2	4.8	2	4.8	4	9.5	19.950	< 0.001
Normal	0	0.0	36	85.7	36	85.7		
High	0	0.0	2	4.8	2	4.8		
Total	2	4.8	40	95.2	42	100,0		

Weight/height initial	Weight/height final						Total		Person’s Chi-square	Asymptotic significance (bilateral)
	Normal		Overweight		Obese					
	f	%	f	%	f	%	f	%		
Malnourished	1	2.4	0	0.0	0	0.0	1	2.4	33.882	< 0.001
Normal	32	76.2	1	2.4	0	0.0	33	78.6		
Overweight	4	9.5	1	2.4	0	0.0	5	11.9		
Obese	0	0.0	1	2.4	2	4.8	3	7.1		
Total	37	88.1	3	7.1	2	4.8	42	100.0		

Percentages may not add up to 100 due to rounding.

### 3.3 Dietary patterns

Table 4 shows the dietary patterns at the beginning and end of the nutritional program in which there is an adequate consumption of protein foods such as eggs between 3 and 4 times a week (78.5 and 81%), foods rich in protein and iron such as legumes between 3 and 4 times a week (71.5 and 69.1%) and a high proportion of meat and fruit consumption on a daily basis in 90.5% at both times.

**Table 4:** Dietary patterns in children under 6 years of age at the beginning and end of the BQDEI consumption within a nutritional program.

Dietary patterns		Nutritional program			
		Initial		Final	
		f	%	f	%
Cereals roots tubers	Daily	32	76.2	33	78.6
	2 t/week	1	2.4	0	0.0
	3 t/week	0	0.0	3	7.1
	4 t/week	2	4.8	0	0.0
	5 t/week	7	16.7	6	14.3
Legumes and nuts	Daily	3	7.1	3	7.1
	1 t/week	1	2.4	1	2.4
	2 t/week	7	16.7	7	16.7
	3 t/week	17	40.5	17	40.5
	4 t/week	13	31.0	12	28.6
	5 t/week	1	2.4	2	4.8
Dairy products	Daily	5	11.9	5	11.9
	1 t/week	1	2.4	1	2.4
	2 t/week	12	28.6	11	26.2
	3 t/week	17	40.5	18	42.9
	4 t/week	5	11.9	5	11.9
	5 t/week	1	2.4	1	2.4
	6 t/week	1	2.4	1	2.4
Eggs	1 t/week	1	2.4	1	2.4
	2 t/week	5	11.9	4	9.5
	3 t/week	24	57.1	22	52.4
	4 t/week	9	21.4	12	28.6
	5 t/week	2	4.8	2	4.8
	6 t/week	1	2.4	1	2.4
Meat and red viscera	Daily	38	90.5	38	90.5
	3 t/week	2	4.8	2	4.8
	4 t/week	1	2.4	0	0.0
	5 t/week	0	0.0	1	2.4
	6 t/week	1	2.4	1	2.4
Fruit and vegetables	Daily	38	90.5	38	90.5
	2 t/week	0	0.0	0	0.0
	5 t/week	3	7.1	2	4.8
	6 t/week	1	2.4	2	4.8
Total		42	100.0	42	100.0

Percentages may not add up to 100 due to rounding.

### 3.4 Presence of intestinal parasites

Table 5 shows that parasites were present in 11.9% of children at the start of the nutritional program, 9.5% at the first follow-up and 7.0% at the final follow-up. *Entamoeba coli*, *Blastocystis hominis*, and *Giardia lamblia* cysts were not present at the first or second follow-up appointments, except for *G. lamblia* in one child. *Enterobius vermicularis* eggs were also observed, with a higher frequency in four children at the second follow-up.

**Table 5:** Presence of intestinal parasites in children under 6 years of age who participated in the nutritional program.

Parasitosis	Basal		1^st^ follow-up		2^nd^ follow-up	
	f	%	f	%	f	%
Positive	5	11.9	4	9.5	3	7.1
*Entamoeba coli*	2	4.8	0	0.0	0	0.0
*Enterobius vermicularis*	1	2.4	4	9.5	2	4.8
*Blastocystis hominis*	1	2.4	0	0.0	0	0.0
*Giardia lamblia*	1	2.4	0	0.0	1	2.4
Negative	37	88.1	38	90.5	39	92.9
Total	42	100.0	42	100.0	42	100.0

Percentages may not add up to 100 due to rounding.

### 3.5 Ferritin and hemoglobin concentration

Table 6 shows the ferritin concentrations of the children in the basal, intermediate and final stages of BQDEI consumption, with an increasing trend and in which there is a very significant increase of approximately 13.5 ng/ml in the ferritin concentration in the final stage compared to the measurement made before the application of the nutritional program.

**Table 6:** Ferritin concentration in children under 6 years of age in the basal, intermediate and final stages of the BQDEI consumption.

Stages	*n*	Ferritin (ng/ml)		Friedman test	Cohen’s d	Kendall’s W
		Mean ± SD (95% CI)	Median (IQR)	Significance (*p*)		
Basal	42	40.67 ± 25.20 (32.81–48.52)	34.70 (24.76)	0.001*	0.44	0.23
Intermediate	42	49.37 ± 22.41a* (42.39–56.36)	42.04 (30.27)			
Final	42	54.19 ± 35.38a* (43.17–65.21)	48.76 (26.21)			

IQR is interquartile range. *p < 0.05 is considered significant; (a) indicates comparison with baseline using the Wilcoxon test.

However, according to Cohen’s d and Kendall’s W values, the effect size is small when comparing the means and medians of baseline and final ferritin concentrations, respectively. Likewise, no significant difference was observed in ferritin concentration between the intermediate and final stages, as determined by the Wilcoxon test (Supplementary Table S5).

At the beginning, 8 children (19%) were identified with basal ferritin below the normal limit of 20 ng/ml and at the end of the nutritional program only one child (2.4%) was identified below this limit (Supplementary Table S6), which is related to a significant improvement in the average ferritin concentration in the final stage.

Table 7 shows a slight increase of approximately 0.3 g/dl in the Hb concentration of children between the baseline and final stages of the nutritional program; however, this change was not statistically significant (*p* > 0.05). No significant difference was observed in mean Hb concentration when comparing across the three time points (Supplementary Table S7).

**Table 7:** Hemoglobin concentration in children under 6 years of age in the basal, intermediate and final stages of the BQDEI consumption within the nutritional program.

Stages	*n*	Hemoglobin (g/dl)		Friedman test
		Mean ± SD (95% CI)	Median (IQR)	Significance (*p*)
Basal	42	11.71 ± 0.91 (11.42–11.99)	11.55 (1.00)	0.283
Intermediate	42	11.83 ± 0.88 (11.56–12.10)	11.90 (1.32)	
Final	42	11.99 ± 1.03 (11.68–12.31)	12.00 (1.13)	

IQR is interquartile range.

At the start of the program, there were 11 children with mild anemia (26.2%) and 3 with moderate anemia (7.1%). Of these, at the final stage of the program, 1 had mild anemia (2.4%) and 1 had moderate anemia (2.4%), respectively. Likewise, an increase in children without anemia was observed from 66.7 to 85.7% between the baseline and final stages (Supplementary Table S8). This qualitatively demonstrates that there was a significant recovery from anemia despite a non-significant increase in Hb concentration in the entire participating group.

## 4 Discussion

As shown in Table 2, factors traditionally associated with anemia, such as low household income, limited access to basic services (potable water and sewage systems) and predominantly secondary-level parental education, are less prevalent in this sample as most participants reside in urban areas. Nevertheless, the literature indicates that these determinants are more prevalent and have a greater impact in regions with disadvantaged socioeconomic conditions. For example, Rahayuwati et al. (67) in their research mentioned that the socioeconomic characteristics of families in West Java, Indonesia, have a significant influence on the eating habits of children under 5 years of age, in which stunting and the presence of anemia are related to the mother’s unemployment and not to the family economy as a whole. Similarly, Al Kaabi et al. (68) in children under 4 years of age in the Iraqi city of Kut, living in rural locations, have lower Hb levels, which was exacerbated when the mother had only elementary education and low socioeconomic status. Sunuwar et al. (69), reported that in children under 59 months of age from 6 Asian countries, 57.3% were anemic because the mother also had the same disease, which led to a growth deficit. A common characteristic of the mothers was that they came from poor homes with a lack of basic services for proper nutrition.

Table 3 shows that the anthropometric parameters height-for-age and weight-for-height improved significantly (*p* < 0.001) following the consumption of BQDEI as part of the nutritional program. These results are consistent with other studies (70–73) that have evaluated the impact of iron-fortified food consumption on child growth and improvement of anthropometric parameters. However, a study by Kowalski et al. (74), found that consuming drinks fortified with multiple micronutrients significantly improved Hb levels and reduced the prevalence of anemia in children, although it had no significant effect on their growth. Anthropometric growth outcomes are influenced by socioeconomic conditions and baseline nutritional status. Evidence suggests that micronutrient fortification alone may be insufficient to improve these indicators unless implemented within a comprehensive framework that includes dietary counseling (72, 75). This study integrated the consumption of BQDEI into a nutritional intervention aimed at promoting adherence to the fortified beverage and adoption of healthy eating habits among children. This intervention was primarily supported by their immediate environment, particularly maternal involvement. The programme incorporated structured activities that positively impacted anthropometric outcomes, highlighting the importance of environmental engagement in medium- and long-term nutritional strategies (30).

The findings reinforce the importance of iron fortification in nutrition programmes targeting vulnerable populations, particularly those affected by nutritional deficiencies or endemic malaria (76, 77). In such contexts, BQDEI may be an effective way to improve nutritional status in cases of malnutrition. The observed increase in height-for-age suggests a beneficial effect on linear growth, with potential long-term implications for child health and development. However, the effectiveness of food fortification is influenced by sociocultural factors that impact dietary practices and health conditions within target populations.

Furthermore, improvements in height-for-age and weight-for-height indicators among children consuming BQDEI may be partially attributed to elevated levels of insulin-like growth factor 1 (IGF-1), a peptide regulated by hormonal and nutritional inputs. Previous studies have shown that protein-rich diets, such as those incorporating quinoa, can enhance IGF-1 concentrations (78). Given its role in promoting bone growth and weight gain, IGF-1 serves as a complementary biochemical marker to anthropometric assessments (79), although it was not directly measured in the present study.

Table 4 shows that the children evaluated have adequate dietary patterns in terms of the frequency of consumption of protein foods rich in iron, such as meats and legumes, as well as vegetables, and that these dietary patterns did not undergo significant changes after the implementation of the nutritional program. There is evidence that the consumption of foods rich in heme iron such as red meat, poultry, viscera and fish, in addition to the consumption of fruits and vegetables rich in folic acid and vitamin C are associated with less anemia in children (80). However, in the present study, the adequate patterns in the participants did not vary at baseline and at the end of the program; so the increases in Hb and ferritin in children are mainly due to the most immediate dietary sources of iron with formulations that allow for better bioavailability (76), which can be achieved with fortified foods, such as the drink used in the PFP.

Data from Table 5 indicate that the prevalence of parasites was higher in children prior to the initiation of the nutritional program. At this stage, cysts of *Entamoeba coli*, *Blastocystis hominis*, and *Giardia lamblia* were identified. Additionally, *Enterobius vermicularis* eggs were detected in all three evaluations, with a particularly elevated frequency observed during the second follow-up. These parasites are generally found on fruits and vegetables due to contamination with cysts (81). The parasite frequencies observed in this study are lower compared to those reported in Argentina, where 54% of the studied child population presented enteroparasites, with the most frequent being *Blastocystis* spp., *Giardia intestinalis*, and *Ascaris lumbricoides* (82). In the present investigation, Ascaris lumbricoides was not detected, but *Enterobius vermicularis* was identified. Furthermore, the findings differ from another study conducted in Argentina, where *E. coli* was more frequently found in fecal samples than other *Entamoeba* species, such as *E. histolytica* and *E. dispar* (83).

On the other hand, it has been reported that, worldwide, approximately 400 million people are affected by *Enterobius*, especially children in developing countries. *Blastocystis* sp. affects one billion people and *Giardia lamblia* affects 200 million. The prevalence of these parasites is higher in temperate climate regions and has much to do with personal hygiene, type of housing, lack of drinking water and sewage service, bed sharing, overcrowding in the home, living in a non-urban area, and poor access to health education and diagnosis and treatment of these parasites (84–86). In this study, although the frequency of parasitosis is low, it may be related to the 11.9% lack of drinking water and 14.3% lack of sewage in the children’s homes.

Although *E. coli* is considered as a non-pathogenic protozoan, which does not present symptoms in infected persons, it must be taken into account that infection by *E. coli* and *Giardia lamblia* can cause malabsorption and promote a proinflammatory response and therefore has long-term consequences for human health. On the other hand, *E. coli* infection can alter the homeostasis of the microbiota causing changes in appetite and metabolism (87), whereas *Enterobius vermicularis* can cause the formation of granulomas in the kidney, in the peritoneal cavity in the female genital tract, the male urinary tract and in the appendix (88, 89). For this reason, although the parasitosis was found in low percentage during the development of the program, it was treated with broad-spectrum antiparasitics to reduce its influence on the results.

Table 6 shows the increase in ferritin concentration observed in participants who consumed the drink proposed in this study. Although a direct comparison with other interventions is not included, it is relevant to contextualize these findings within the existing literature. For example, Kowalski et al. (74) reported an increase of approximately 26 ng/ml in children in a rural area of Guatemala after administration of a drink fortified with multiple micronutrients (MMN), compared to an increase of approximately 20 ng/ml in the placebo group. In this study, the increase was approximately half that reported in Guatemala. This difference could be related to the fact that most of the participants belong to urban areas, despite the fact that both studies applied a similar treatment time and worked with child populations of comparable age.

The positive variation of ferritin up to either the intermediate or final stage with BQDEI is higher than that determined in a meta-analysis by Aaron et al. (87), who found that in school-aged children from lower-middle income countries, ferritin levels improve with an increase of 6.86 ng/ml when given non-dairy drinks fortified with MMN for a period of 8 weeks to 6 months.

In a setting closer to our reality in Viçosa, a city in the state of Minas Gerais in Brazil, they also developed a study with fortified milk powder drink (89) that included vitamin A, iron in the form of ferrous fumarate, zinc and copper in the form of sulfate and prebiotics, administered to preschool children, but in that study the increase in ferritin was approximately twice as high as that found in the present work. This is mainly due to the addition of the added nutrients that add to the effect established by the iron provided in the product. With regard to BQDEI, it could only have an effect on the increase in ferritin, iron in the form of FPP, organic acids, and vitamin C (the latter two promote iron absorption); however, its use may exceed its effect on ferritin concentration compared to fortified milk drinks, because milk as a vehicle has some inhibitory effects on iron absorption due to casein and whey proteins (90).

In subjects with sufficient iron, there is a decrease in the expression of divalent metal transporter-1 (DMT-1), so it is considered a limiting factor, important in the absorption of this metal as it reduces the differences in absorption in the face of variable solubility of iron compounds. Although duodenal cytochrome b expression is not down-regulated, it allows the available ferric iron to be converted to the ferrous form for absorption. In iron deficiency, there is an increased expression of DMT1 on the apical surface of the microvilli of the intestine and in this condition, solubility is the main limiting factor in iron absorption. At the same time, duodenal cytochrome b is not up-regulated in its expression. Therefore, it favors even more the absorption of ferrous iron over ferric iron (91). It appears that in children receiving BQDEI, an increased activity of duodenal cytochrome b was mainly established to favor the absorption of iron from FPP.

A low serum ferritin concentration suggests deficient iron stores, whereas elevated levels are indicative of iron overload, but also an infectious or inflammatory process. However, low iron stores are considered the first stage of a process that can lead to iron deficiency anemia (92). Clearly, in the present study, the increase in ferritin concentration by 13.5 ng/ml demonstrates storage of absorbed iron overload, enough to prevent anemia in children and maintain normal Hb concentration.

The slight increase in Hb after consumption of BQDEI is similar to the study by Kowalsky et al. (74), where the drink “Chispuditos” showed no significant differences in Hb between groups. In contrast, Vuvor et al. (93) reported significant increases with cereals fortified with ferrous fumarate (1.97 g/dl in the intervention group). Csölle et al. (76), also showed substantial improvements in Hb (3.44 g/L) and a lower prevalence of anemia with fortified foods. Finally, Castro et al. (89), achieved a 0.42 g/dl increase in Hb with a fortified powdered prebiotic, showing results comparable to BQDEI, supported by a robust methodological design.

The results may differ due to the fortifier used in the aforementioned studies, such as inorganic and organic ferrous salts with higher iron bioavailability for the body (33, 94). On the other hand, iron pyrophosphate is an interesting alternative for cases where tolerance and stability are priorities, although it may be less efficient compared to more bioavailable forms such as ferrous sulfate (95) or ferrous ascorbate (96). However, studies have been conducted with different forms of FPP to improve its bioavailability and compare it with ferrous sulfate, with similar results in both ferritin and Hb with the use of the micronized form (34) and liposomal form (97).

In the present study, a form of FPP stabilized with amino acids was used as a fortifying agent in blueberry and quinoa drinks. Although no significant increase in average Hb was observed after the intervention, analysis of frequencies and proportions using the McNemar test showed a significant reduction in the prevalence of anemia (Supplementary Table S6). Similar results were reported by Radhika et al. (98), who used micronized ferric pyrophosphate (MFPP) in extruded rice grains incorporated into a rice-based meal, obtaining positive effects in children aged 5 to 11 years in a control group design.

However, the complementary finding observed in the present study should be interpreted with caution, as the pre-experimental design without a control group limits the ability to attribute the changes exclusively to the intervention. In addition, external variables could not be fully controlled, such as factors inherent to the product, including the presence of phenolic compounds that affect iron absorption, the specific bioavailability of FPP as a fortifier, and possible food or drug interactions with the iron in the product that are difficult to evaluate in the field (94) which is why a weak effect of BQDEI on ferritin and Hb concentration was obtained.

There is evidence that the consumption of fortified complementary foods significantly reduced the likelihood of children developing anemia (99, 100), provided that nutritional education, dietary monitoring, and parasitic control are implemented within a nutritional program, as demonstrated in the present study.

One of the limitations of the study was the absence of a control group, because it was decided to give the same treatment to the participants who accepted the study. Likewise, it was not planned to elaborate a control product, with similar characteristics to that of the BQDEI, due to the production schedules of the plant of the agro-industrial company where the work was coordinated as a whole.

Although complementary actions were carried out, such as monitoring nutrition, controlling parasitic infections, and providing guidance on the adequate consumption of iron-enriched cranberry and quinoa drinks, these measures do not replace the methodological rigor provided by a control group. Therefore, the results should be interpreted with caution, due to other uncontrolled factors that may have influenced the observed effects and which have been discussed above. However, as blueberries have a low iron content in the formulation, approximately 0.28 mg/100 g of edible portion of the fruit (37), it is considered that the positive effect observed in ferritin concentration and, to a lesser extent, in Hb, is due to the fortification provided in the drink with ferric pyrophosphate, in addition to the fact that their diet remained constant during the nutritional program.

Another important limitation is the non-adherence at the beginning of the intermediate stage of the study, which represented 22%. However, this aspect was improved with recommendations by the nutritionists for strategies regarding the ways of how to consume the BQDEI and the times of such consumption, to ensure the completion of the children’s participation in the research.

Preliminary results support the potential of BQDEI as a functional food in complementary feeding strategies, and longitudinal studies are recommended to establish a causal relationship between consumption of the drink, combined with monitoring of protein food consumption and IGF-1 modulation in contexts of prevention of anemia and child malnutrition.

For ethical reasons, all children were considered regardless of their Hb level, which was a slight disadvantage since most of the children had normal Hb levels (see Supplementary Table S8). For that reason, a slight increase in this parameter was evident and therefore not significant after the BQDEI. Nevertheless, the results indicate that, if developed in other settings where malnutrition and iron deficiency exist, the BQDEI would have a great impact not only in the prevention of anemia by its positive influence on ferritin concentration, but also in the increase of Hb. Likewise, the BQDEI could be used before and during pregnancy to promote iron storage in the fetus, which would be preventive even in preterm infants who are more likely to develop iron deficiency anemia (101).

As a strength, we consider the work of two groups linked in the research, one by the technological development of the product by the Company with the sanitary guarantees and also of the standardized chemical composition, and another by the University researchers in the orientation of the feeding and monitoring of the consumption of the product, in which the only interest is to contribute to the solution of a relevant social problem such as anemia in children, without economic benefits and contributing to the solution of the Sustainable Development Goal (SDG) 3 that refers to health and well-being (102). The results of the research were obtained by an external and certified laboratory, so it is assumed that there was no manipulation of any kind by the researchers.

It would be advisable to add other nutrients to the BQDEI to verify a significant increase in Hb concentration in relation to that obtained in the present investigation. Likewise, its combination with iron in the ferrous form should also be considered in drinks with FPP, to increase the absorption of iron in children from areas more vulnerable to anemia, due to the fact that there is a greater expression of the DMT-1 in those children (91).

## 5 Conclusion

It was demonstrated that the nutritional program based on the consumption of the BQDEI added to the educational sessions and nutritional follow-up can prevent anemia in children, slightly improve nutritional status and increase the concentration of Hb and ferritin, being more significant in ferritin. This program can be applied in children in regions where anemia is more prevalent.

After validating BQDEI as a preventive strategy for anemia within the framework of a nutrition program, the formation of strategic alliances is proposed to scale this initiative to the national level. The objective is to expand its reach to more families and communities in Peru, thus promoting a replicable model with the potential for a direct impact on the sustained reduction of anemia. This proposal seeks to contribute to strengthening the country’s productivity, human development, and competitiveness through an innovative, evidence-based nutritional intervention.

## Data Availability

The original contributions presented in the study are included in the article/Supplementary material, further inquiries can be directed to the corresponding authors.
