# Supplementary material for: Prevention of anemia in children through the consumption of a blueberry and quinoa drink enriched with iron as part of a nutritional program
Source: Front Nutr. 2025 Sep 26;12:1639894. doi: 10.3389/fnut.2025.1639894 (PMC12510819; doi:10.3389/fnut.2025.1639894)
Supplement: Supplementary file 1 [file Table_1.DOCX]

#
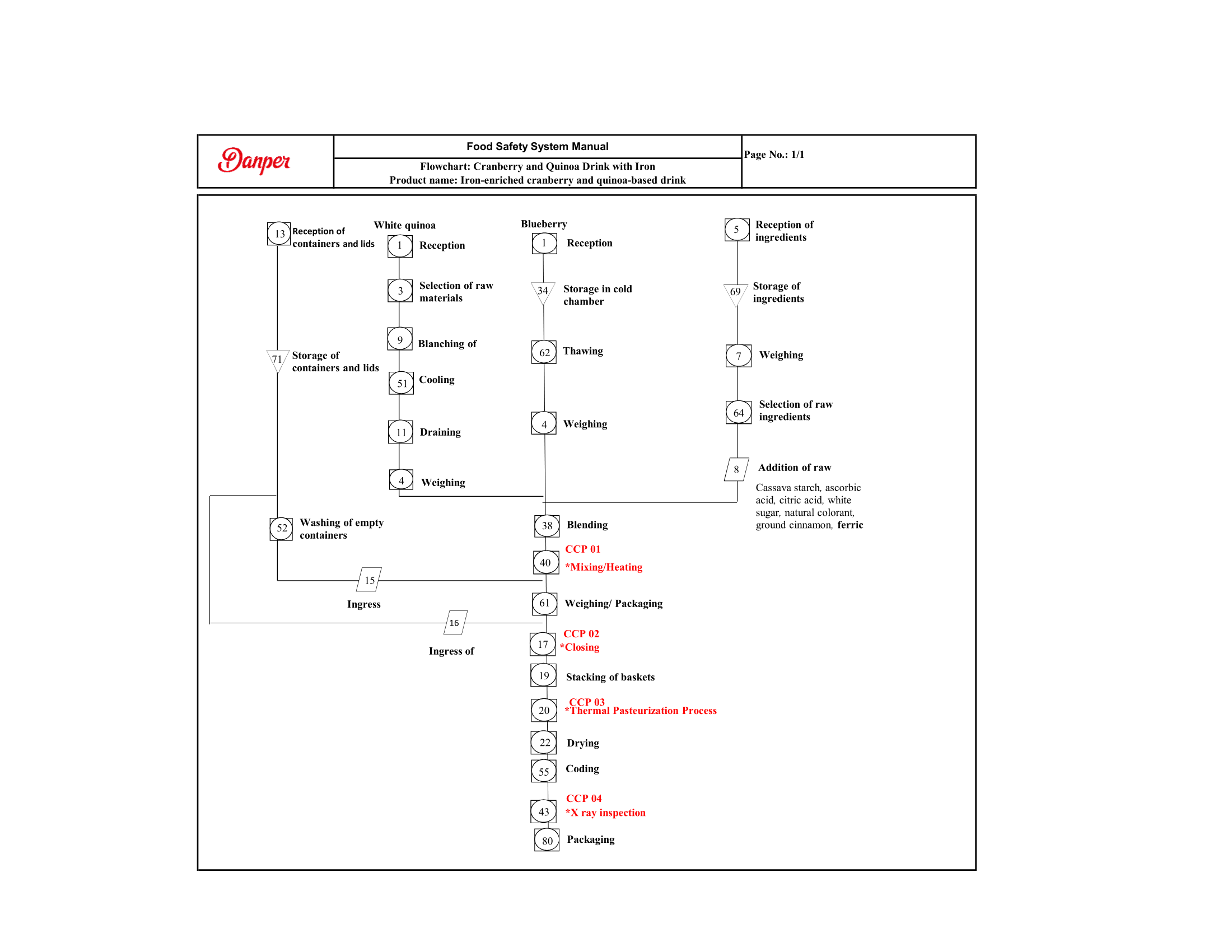


# Figure S1. Flow chart of the production of cranberry and quinoa drink enriched with ferric pyrophosphate

# Instrument S1. Format for sensory assessment in children under 6 years old

**
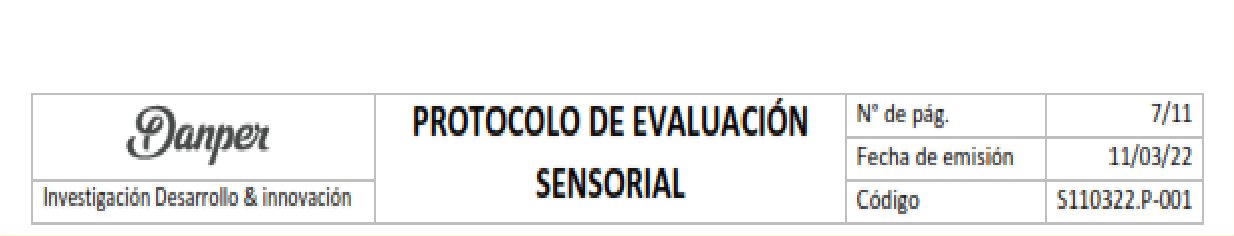
**

**Preference Assessment Form - Children Name: .............................................. Age: .... Date: ..../..../ /**

**Instructions: Please test the product and mark the face according to the level of preference you feel is appropriate.**

**Product: Blueberry drink with quinoa**

**
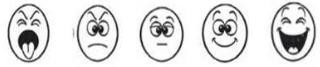
**

| I hate it  (1) | I didn't like it  (2) | Indifferent  (3) | I liked it  (4) | I loved it  (5) |
| --- | --- | --- | --- | --- |

# Instrument S2. Data collection sheet General data

Child: Age: Sex:

# Socio-economic data:

- 1. Family income
     1. less than 500 soles b) 500 - 1050 soles c) 1050 - 2000 soles d) more than 2000 soles
  2. Occupation
     1. Independent b) dependent c) housewife d) other:
  3. Marital status
     1. Single b) Married c) Cohabiting d) Separated e) Widowed
  4. Level of education
     1. Illiterate b) Elementary School c) High School d) Higher Education
  5. Origin
     1. rural b) urban
  6. Number of members
     1. less than or equal to 4 (b) greater than 4
  7. Basic services

Access to water: a) Yes ( ) b) No ( ) Access to drainage: a) Yes ( ) b) No( ) Access to light: a) Yes ( ) b) No ( )

# Anthropometric data

For children

Weight: Height:

Height/age: Weight/height: Weight for age:

# Hemoglobin

Concentration (g/dL):

a) normal b) mild anemia c) moderate anemia d) severe anemia

# Ferritin

Concentration (g/dL):

a) < 20 ng/ml b) ≥ 20 ng/ml

# IV. Parasitosis

Presents: a) Yes b) No

Parasite identified: ................................

# Instrument S3

**Frequency of consumption of food groups for children under 6 years of age.**

| **Group** | **Group Food that compose it** | **Diary** | **Weekly** | **Monthly** | **Remarks** |
| --- | --- | --- | --- | --- | --- |
| **Group 1** | Cereals, roots Tubers |  |  |  |  |
| **Group 2** | Legumes and nuts |  |  |  |  |
| **Group 3** | Dairy products (milk, cheese, yoghurt) |  |  |  |  |
| **Group 4** | Eggs |  |  |  |  |
| **Group 5** | Meat (including poultry and fish) red viscera |  |  |  |  |
| **Group 6** | Fruits and vegetables dark green, orange, red (rich in Vitamin A) |  |  |  |  |
| ***Adapted from "Dietary Diversity as a Measure of the Micronutrient Adequacy of Women's Diets: Results from Rural Bangladesh Site - FANTA".** | | | | | |

**Table S1** Anthropometric classification for male children under 5 years of age

| Anthropometric table for male children under 5 years of age | | |
| --- | --- | --- |
| Weight for age classification | Severe malnutrition | < to weight at -3 SD * |
|  | Malnourished | ≥ to weight at -3 SD |
|  | Normal | between - 2 SD and 2 SD |
|  | Overweight | ≤ to weight at 3 SD |
|  | Obesity | > to weight at 3 SD |
| Height for age classification | Severe low height | < to height at -3 SD |
|  | Low height | ≥ to height at -2 SD |
|  | Normal | between - 2 SD and 2 SD |
|  | High height | > to height at 2 SD |

* Standard deviation (SD)

**Table S2** Anthropometric classification for female children under 5 years of age

| Anthropometric table for female children under 5 years of age | | |
| --- | --- | --- |
| Weight-height classification | Severe malnutrition | < to weight at -3 SD |
|  | Malnourished | ≥ to weight at -3 SD |
|  | Normal | between - 2 SD and 2 SD |
|  | Overweight | ≤ to weight at 3 SD |
|  | Obesity | > to weight at 3 SD |
| Height for age classification | Severe low height | < to height at -3 SD |
|  | Low height | ≥ to height at -3 SD |
|  | Normal | between - 2 SD and 2 SD |
|  | High height | > to height at 2 SD |

**Table S3** Anthropometric classification for boys and girls over 5 years of age

| **Anthropometric table for boys over 5 years of age** | | |
| --- | --- | --- |
| **Body mass index (BMI) for age** | Thinness | < -2 SD |
|  | Normal | between ≥ -2 SD and -1SD |
|  | Overweight | ≤ 2 SD |
|  | Obesity | > 2 SD |
| **Height for age** | Low height | < to height at -2 SD |
|  | Normal | ≥- 2 SD between ≤ 2 SD |
|  | High height | > to height at 2 SD |

**Table S4** Classification of anemia according to age

| **Technical standard** | **Age** | **Anemia (g/dL) according to age** | | | |
| --- | --- | --- | --- | --- | --- |
|  |  | **Severe** | **Moderate** | **Mild** | **Without anemia** |
| **Health Technical Standard**  **Anemia Prevention and Control 2017 ^(62)^** | From 6 months to 5 years old | < 7.0 | 7.0 - 9.9 | 10.0 - 10.9 | ≥ 11.0 |
| **Health Technical Standard**  **Anemia Prevention and Control 2024 ^(63)^** | 6 to 23 months | < 7.0 | 7.0 - 9.4 | 9.5 - 10.4 | (≥ 10.5 |
|  | 24 to 59 months | < 7.0 | 7.0 - 9.9 | 10.0 - 10.9 | ≥ 11.0 |
|  | 5 to 11 years old | < 8.0 | 8.0 - 10.9 | 11.0 - 11.4 | ≥ 11.5 |

**Table S5** Wilcoxon signed-rank test results comparing ferritin concentrations across different stages of the nutritional program

| **Mean Ferritin Comparison**  **(Time Points)** | **Wilcoxon Test**  **(Z)** | **Significance**  **(p)** |
| --- | --- | --- |
| **Baseline Ferritin – Intermediate Ferritin** | -2.945 | 0.003* |
| **Baseline Ferritin – Final Ferritin** | -3.157 | 0.002* |
| **Intermediate Ferritin – Final Ferritin** | -0.569 | 0.569 |

**Note: *p<0.05 es significant.**

**Table S6** Ferritin concentration in children under 6 years of age before and after the BQDEI consumption.

| **Ferritin concentration** | | **After** | | | | **Total** | | **Significance**  **(p)** |
| --- | --- | --- | --- | --- | --- | --- | --- | --- |
|  |  | < 20 ng/mL | | ≥ 20 ng/mL | |  |  |  |
|  |  | **f** | **%** | **f** | **%** | **f** | **%** |  |
| **Before** | < 20 ng/ml | 1 | 2.4 | 7 | 16.7 | 8 | 19.0 | 0.008* |
|  | ≥ 20 ng/ml | 0 | 0.0 | 34 | 81.0 | 34 | 81.0 |  |
| **Total** | | **1** | **2.4** | **41** | **97.6** | **42** | **100.0** |  |

**Note:** **McNemar Exact test was used. *p<0.05 is significant.**

**Table S7** Wilcoxon signed-rank test results comparing hemoglobin concentrations across different stages of the nutritional program

| **Mean Hb Comparison**  **(Time Points)** | **Wilcoxon Test**  **(Z)** | **Significance**  **(p)** |
| --- | --- | --- |
| **Intermediate Hb – Baseline Hb** | -0.921 | 0.357 |
| **Final Hb – Baseline Hb** | -1.686 | 0.092 |
| **Final Hb – Intermediate Hb** | -0.545 | 0.586 |

**Table S8** Behavior of the presence of anemia in children under 6 years of age before and after consumption of the BQDEI

| **Anemia** | | **After** | | | | | | **Total** | | **Significance (p)** |
| --- | --- | --- | --- | --- | --- | --- | --- | --- | --- | --- |
|  |  | **Mild** | | **Moderate** | | **No anemia** | |  |  |  |
|  |  | f | % | f | % | f | % | f | % |  |
| **Before** | **Mild** | 1 | 2.4 | 0 | 0 | 10 | 23.8 | 11 | 26.2 | 0.046* |
|  | **Moderate** | 1 | 2.4 | 0 | 0 | 2 | 4.8 | 3 | 7.1 |  |
|  | **Severe** | 0 | 0 | 0 | 0 | 0 | 0 | 0 | 0 |  |
|  | **No anemia** | 4 | 9.5 | 0 | 0 | 24 | 57.1 | 28 | 66.7 |  |
| **Total** | | **6** | **14.3** | **0** | **0** | **36** | **85.7** | **42** | **100** |  |

# Note: Cases of anemia were grouped. McNemar's exact 2x2 test was used to compare the "anemia" and "no anemia" groups before and after. *p<0.05 is significant.
